# Inverted Follicular Keratosis in a Young Female Patient: A Case Report of a Rare Occurrence

**DOI:** 10.7759/cureus.77907

**Published:** 2025-01-24

**Authors:** Haifa A Alfalah, Halah O Alamawi, Hamad J Aldhafiri, Alanood M Alharthi

**Affiliations:** 1 Department of Dermatology, King Abdullah bin Abdulaziz University Hospital, Riyadh, SAU; 2 College of Medicine, Princess Noura bint Abdulrahman University, Riyadh, SAU; 3 Department of Dermatology, King Khalid General Hospital, Hafar Al Batin Health Cluster, Hafar Al Batin, SAU

**Keywords:** dermatology, dermato-pathology, differential diagnosis, histopathology, ifk, inverted follicular keratosis, solitary skin lesion, young female

## Abstract

This case report describes a rare occurrence of inverted follicular keratosis (IFK) in a 27-year-old woman who had a single lesion on the left side of her nose. IFK, which primarily affects older males, is a harmless condition that involves the hair follicles and sebaceous ducts. The patient's lesion, with a size of 0.4 x 0.3 x 0.3 cm, was surgically removed and subsequently identified as IFK through histopathological analysis. This case underscores the significance of including IFK in the list of potential causes for solitary keratotic lesions in younger patients and emphasizes the need for histopathological analysis to ensure an accurate diagnosis. After the surgical removal, the patient did not experience any return of the condition, which aligns with the positive outlook described in the medical literature.

## Introduction

Inverted follicular keratosis (IFK), also known as Helwig's inverted follicular keratosis, is a benign and rare disease affecting hair follicles, the infundibulum, and sebaceous duct openings. The lesion is most frequently present as a solitary lesion [1]. IFK is prevalent in elderly and male patients [2] and frequently involves the lips and cheeks [1]. IFK duration is estimated to be between two months and four years in many cases [1]. Moreover, an average lesion measurement of 0.2 cm to 1 cm was observed among cases [3]. Common differential diagnoses include seborrheic verruca, keratoacanthoma, and squamous cell carcinoma [3]. IFK involves multiple known histopathological features like parakeratosis, squamous eddies, pseudo-horn cysts, keratin pearls [1], and basaloid cells [3]. Overall, IFK has a favorable clinical outcome, and none of the cases observed showed recurrence [1,2]. Herein, we present a case report of inverted follicular keratosis in a young female patient where IFK involved the nose.

## Case presentation

A 27-year-old female with a known history of atopic dermatitis and gastroesophageal reflux disease (GERD) presented to our department with a complaint of a solitary, slowly enlarging skin lesion on the left nasal ala, persisting for approximately 11 months. The lesion's gradual growth was initially unnoticed by the patient until she observed its persistence and slight enlargement over time.

Upon further inquiry, the patient reported that the lesion first appeared as a small, asymptomatic nodule, which she initially dismissed as a benign skin anomaly. However, over the past few months, she noticed a gradual increase in its size, prompting her to seek medical advice. The lesion occasionally bled spontaneously, though the patient could not correlate this with any specific triggers such as trauma or scratching. There were no associated systemic symptoms like fever, weight loss, or night sweats.

**Figure 1:**
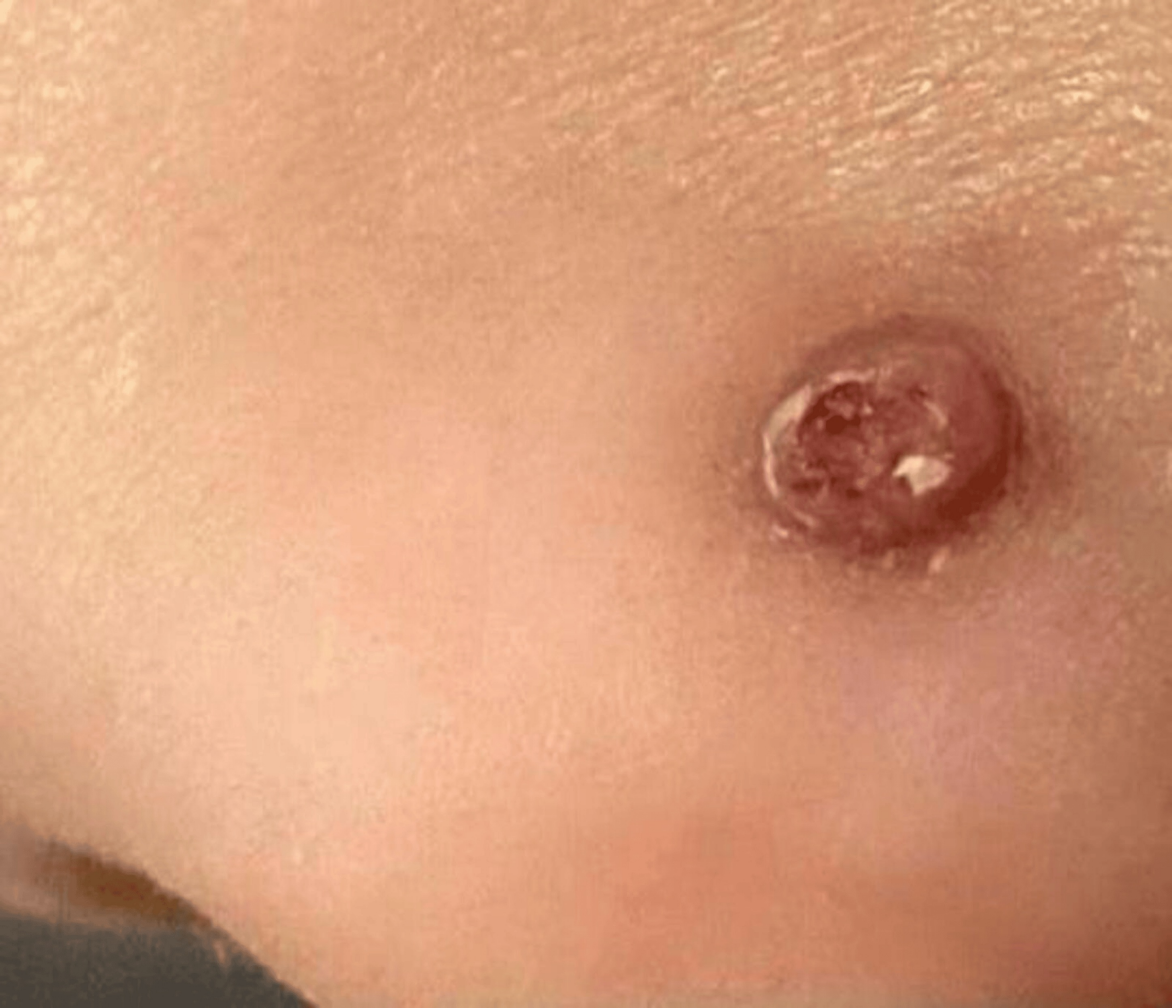
A solitary, dome-shaped, exophytic nodule is observed resembling inverted follicular keratosis.

On dermatological examination, a pedunculated, skin-colored round mass was observed on the left nasal ala, measuring approximately 0.4 x 0.3 x 0.3 cm. The lesion had a slight crustation on its surface, with central ulceration. The surrounding skin exhibited minimal erythema. Notably, there was no evidence of induration, discharge, or surrounding edema. The lesion is shown in Figure 1.

The mass was not associated with pain, numbness, or pruritus, and the patient denied any similar lesions elsewhere on her body. Her medical history was significant for atopic dermatitis, a condition she managed with intermittent use of emollients and topical corticosteroids. However, she was not on any active treatment at the time of presentation. Her GERD was well-controlled with dietary modifications and occasional use of proton pump inhibitors. The patient had no history of smoking, alcohol use, or other risk factors for skin malignancies. Her family history was unremarkable for skin cancers or other dermatological conditions.

**Figure 2:**
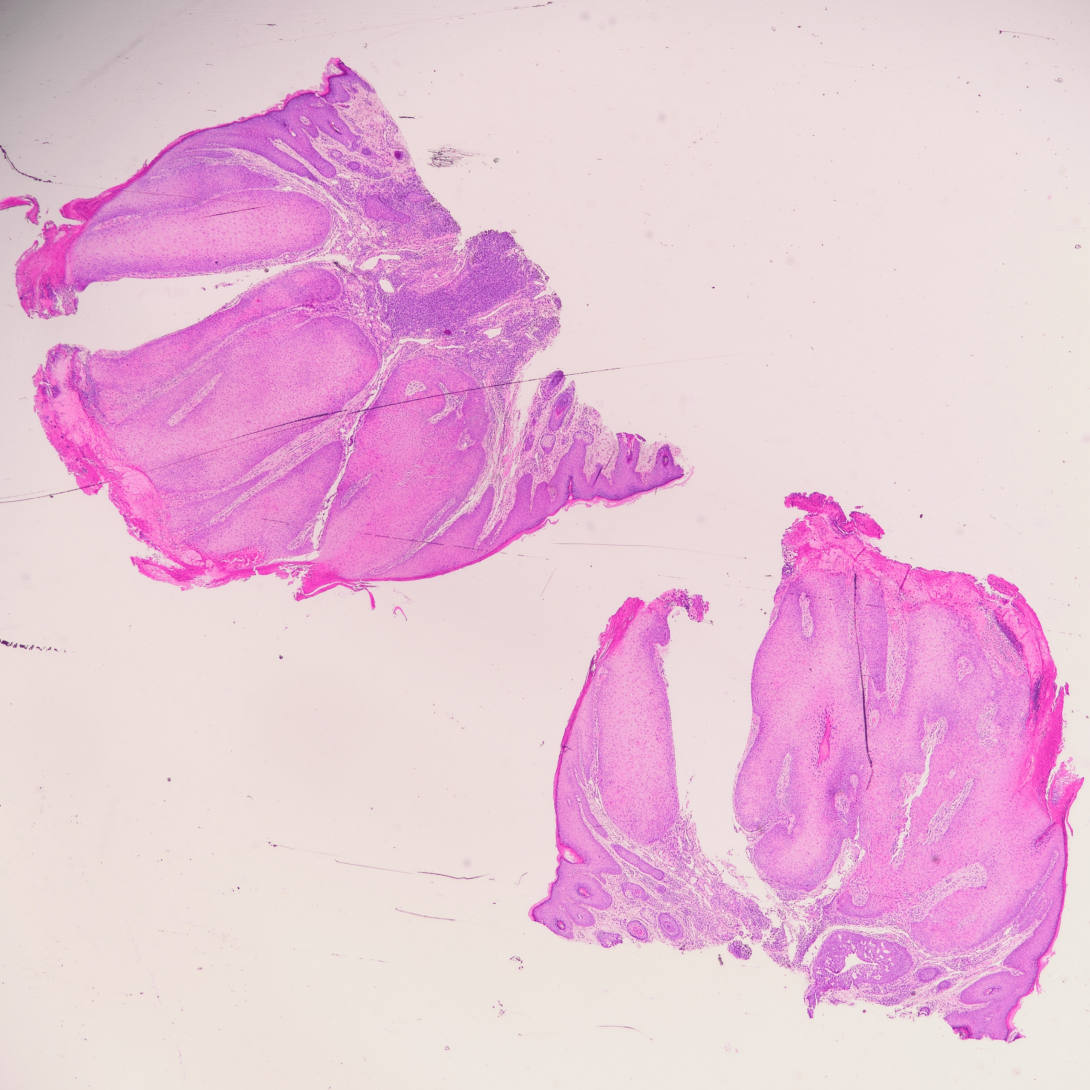
Low power histologic review (H&E) from the skin shows a well circumscribed keratinized squamous cell lesion, with an endophytic growth pattern. (H&E): Hematoxylin and Eosin.

**Figure 3:**
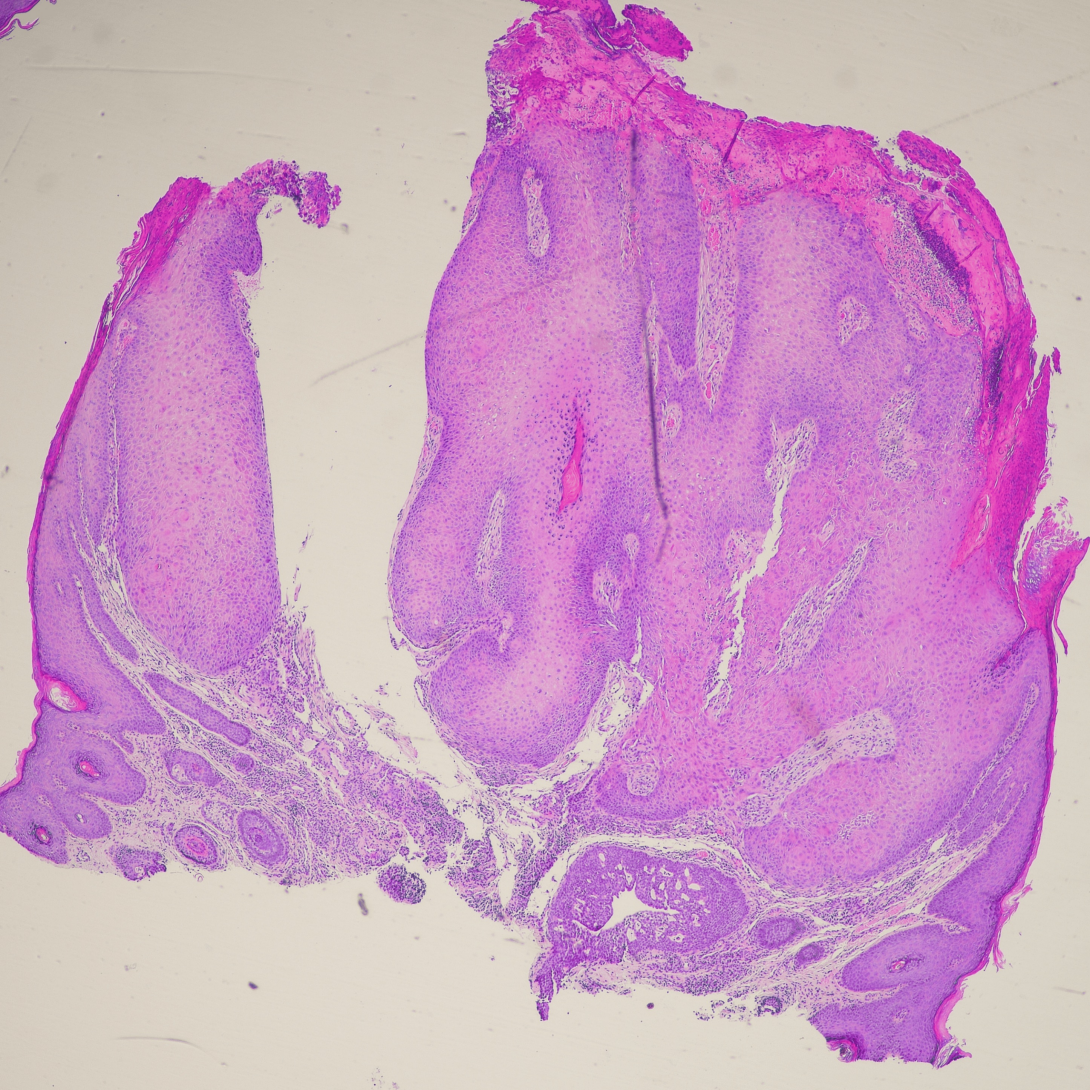
Low power histologic review (H&E) from the skin shows a well circumscribed keratinized squamous cell lesion, with an endophytic growth pattern. (H&E): Hematoxylin and Eosin.

**Figure 4:**
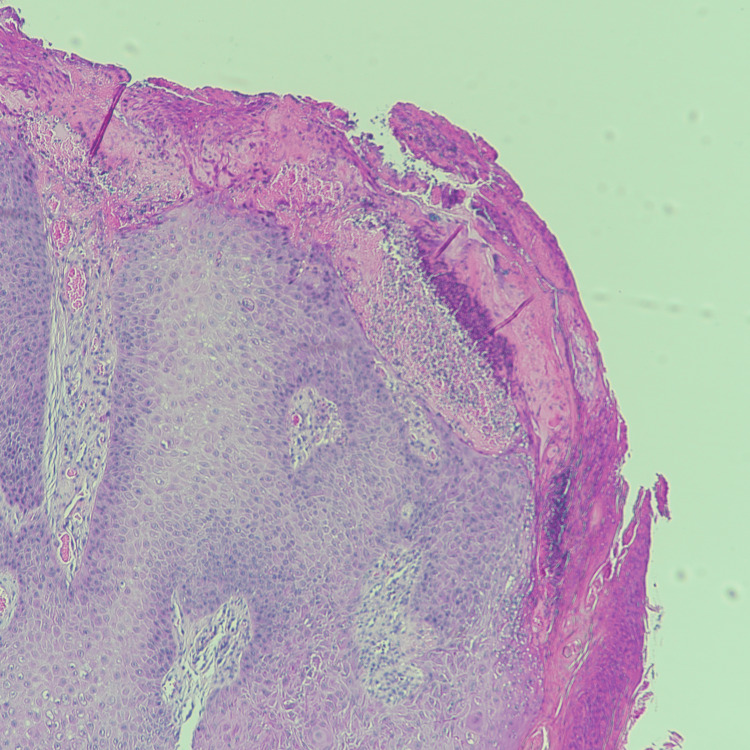
High power histologic review (H&E) shows surface parakeratosis. (H&E): Hematoxylin and Eosin.

**Figure 5:**
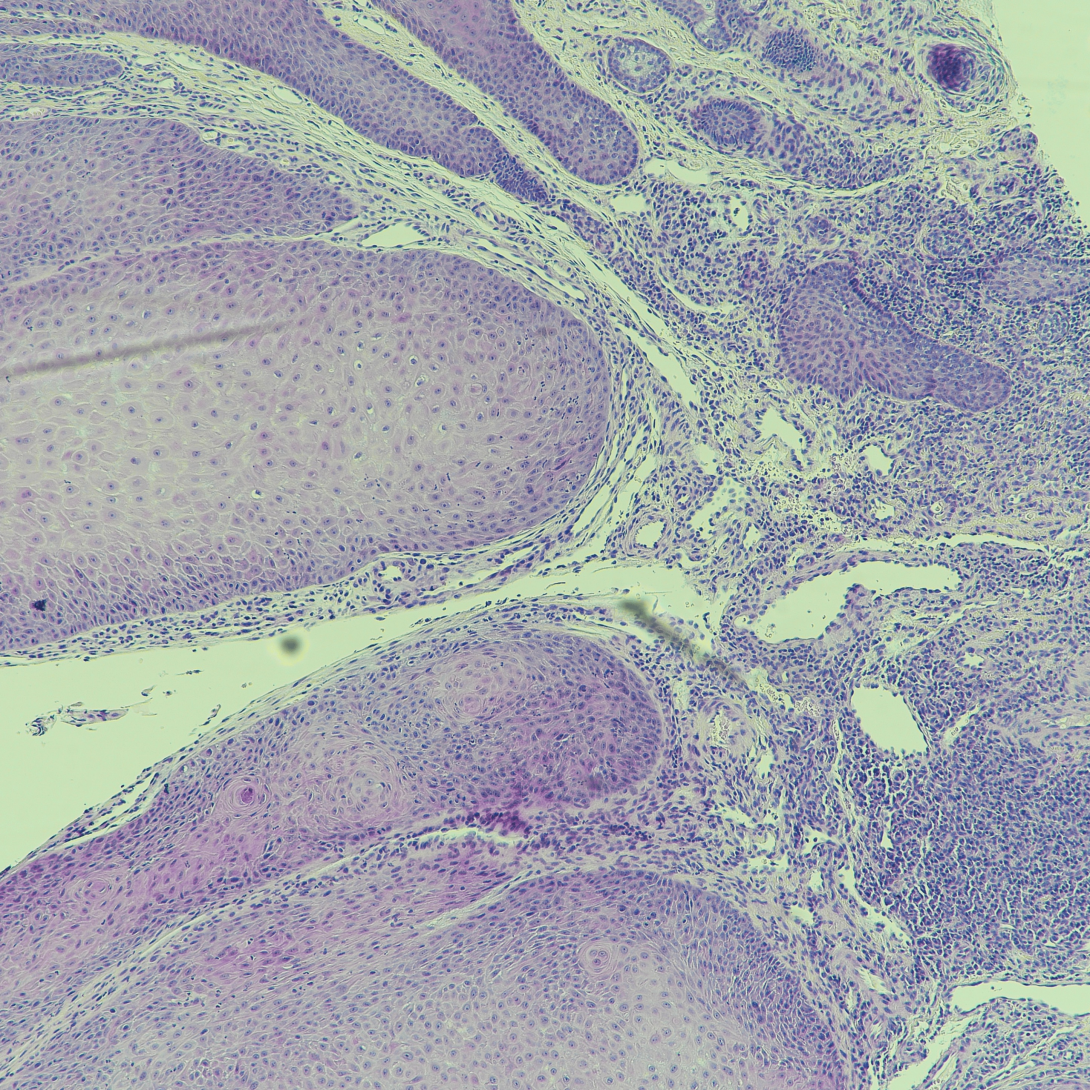
High power histologic review (H&E) demonstrates the inward bulbous growth pattern of this lesion. In addition, squamous eddies and peripheral palisading basaloid cells are noted. (H&E): Hematoxylin and Eosin.

Given the clinical findings, the lesion was excised for histopathological evaluation. The histopathological examination revealed features consistent with parakeratosis, inward bulbous growth, palisading basaloid cells, and squamous eddies (Figures 2-5), suggesting a benign lesion with no evidence of malignancy. The patient tolerated the procedure well, with no immediate postoperative complications. At her follow-up visit, the surgical site had healed appropriately, and there was no evidence of recurrence or new lesions.

## Discussion

Our case report presents an unusual perspective on the occurrence of IFK in a young patient, which is typically observed in older adults. This case highlights the importance of including IFK as part of the differential diagnosis, regardless of the patient's age. This is particularly relevant when dealing with solitary keratotic lesions on sun-exposed areas of the skin [4]. From a clinical perspective, IFK presents as a single, firm papule or plaque. It is a slow-growing lesion that can appear as a filiform verrucous or hyperkeratotic nodular lesion. At times, the lesion may exhibit a smooth surface or possess a central cystic cavity. Located mainly on the face and neck, our patient presented with a pedunculated lesion on the nasal ala. This aligns with the descriptions found in the literature, which state that IFK usually occurs on the face. The diagnosis is typically confirmed through histopathology, as the clinical appearance can be challenging to distinguish from other types of lesions. Its non-specific appearance can lead to confusion with more prevalent dermatological conditions [5,6]. Dermoscopic examination frequently uncovers various patterns. The most prevalent pattern is a keratoacanthoma-like formation consisting of a central keratin surrounded by hairpin vessels arranged radially. In our case, the histopathological examination played a crucial role in diagnosing the condition, confirming that it was IFK rather than a malignant condition, which is often the initial clinical suspicion. The literature affirms the importance of histopathology in differentiating IFK from other keratinizing tumors [7]. Potential differential diagnoses include viral warts, seborrheic keratosis, actinic keratosis, basal cell carcinoma, and squamous cell carcinoma [8]. The literature emphasizes that complete removal of the lesion, as done in this case, is an effective method to prevent the lesion from returning and achieve a permanent cure. Additionally, a 5% imiquimod cream has been reported as an effective treatment for this type of case [8,9]. The favorable result in our case, characterized by the lack of recurrence, corresponds to the documented low rates of recurrence after the removal of the affected area.

## Conclusions

This case demonstrates the crucial importance of conducting a thorough examination of the skin and confirming the diagnosis through microscopic examination of tissue samples in the treatment of IFK. Increasing knowledge about the prevalence of the condition in younger individuals and identifying its specific characteristics when examined under a dermatoscope can assist medical professionals in preventing incorrect diagnoses and providing suitable treatment.
